# Molecular imaging of viral pathogenesis and opportunities for the future

**DOI:** 10.1038/s44303-024-00056-w

**Published:** 2025-01-24

**Authors:** Brianna Kelly, Jeanette E. Boudreau, Steven Beyea, Kimberly Brewer

**Affiliations:** 1 Biomedical MRI Research Laboratory (BMRL), IWK Health Centre, Halifax, NS Canada; 2 Department of Microbiology & Immunology, Dalhousie University, Halifax, NS Canada; 3 Department of Pathology, Dalhousie University, Halifax, NS Canada; 4 Beatrice Hunter Cancer Research Institute (BHCRI), Halifax, NS Canada; 5 IWK Health Centre, Halifax, NS Canada; 6 Department of Diagnostic Radiology, Dalhousie University, Halifax, NS Canada; 7 School of Biomedical Engineering, Dalhousie University, Halifax, NS Canada; 8 Department of Physics & Atmospheric Science, Dalhousie University, Halifax, NS Canada

**Keywords:** Imaging techniques, Molecular imaging, Molecular imaging, Viral tracing, Diseases

## Abstract

Molecular imaging is used in clinical and research settings. Since tools to study viral pathogenesis longitudinally and systemically are limited, molecular imaging is an attractive and largely unexplored tool. This review discusses molecular imaging probes and techniques for studying viruses, particularly those currently used in oncology that are applicable to virology. Expanding the repertoire of probes to better detect viral disease may make imaging even more valuable in (pre-)clinical settings.

## Introduction

Molecular imaging (MI) is a valuable tool for non-invasive diagnostic imaging, treatment response, and study of disease in both clinical and research settings^1^. MI is commonly used in oncology to monitor tumor growth, metastasis, and therapeutic responses. The use of MI to study infectious diseases is becoming more widespread, with imaging being conducted to study viral infection and persistence, as well as immune responses to viruses and other pathogens. It is possible to image both infection and inflammation for viruses using MI. Infection refers to the entry and proliferation of viruses in the host, while inflammation is the immune response and cell recruitment that occurs in response to infection. Both can give valuable information about viral interactions with a host and the pathology that results. Depending on the type of MI and probe used, this technique can be sensitive to changes at a molecular level—though these can only be spatially resolved at a mm or cm macroscopic level. Some of the primary benefits of MI include that it is non-invasive and is suitable for longitudinal studies in a single subject—reducing statistical variation. Several different types of MI exist that look for functional changes^2^.

This work discusses the various MI modalities available for studying viral processes and provides examples of imaging probes that can be used with them. We discuss probes previously used to study the pathology of viruses and those currently in use for cancer that have the potential for translation to virology. Finally, we explore the roles, requirements, and potential for future probes to monitor viral infection based on established viral targets and available imaging technology. While clinical applications of all these probes are possible, some may be limited in widespread implementation by technology availability in clinical settings and the lack of facilities to conduct the necessary radiochemistry. For an in-depth discussion of how each modality works, please refer to the reviews by Prior et al.^3^, Pirovano et al.^4^, and Boros et al.^5^.

### Viral pathogenesis and imaging

While the field of virology has been at the forefront of public health for centuries, the COVID-19 pandemic has re-emphasized the need for novel approaches to the study of viruses. Despite the pandemic bringing on unprecedented scientific milestones in the study of coronaviruses, it also highlighted the lack of tools available to clinicians and researchers to assay the physiological damage created by viral progression. This is not unique to COVID-19: detailed pathogenesis in humans of many viruses remains poorly characterized. Indeed, in existing and emerging virus infections, MI represents a promising technique for studying viruses and their pathology. Pathogenesis includes several factors, such as viral identity, host immune system, and tissue tropism. In essence, this results in all viruses having unique methods of causing disease, the extent of which will vary on an individual host level.

Viral pathogenesis starts at a molecular level, so traditional microbiologic detection and monitoring methods (such as viral culture and immune assays) to study disease processes can often only detect it several steps into the disease. Furthermore, traditional methods of studying viruses tend to be invasive and give a single timepoint snapshot of the disease process when sampling was performed. These methods include infecting cell cultures to look at changes in cytokine secretion, cell morphology, and other biomarkers. The use of humanized animal models is standard in research; however, there remains a lack of longitudinal data and non-invasive techniques^6^. In clinical settings, histological samples of infected tissues can be taken, but this is highly invasive and usually only done at a severe stage of infection and does not capture systemic immune effects^7^. An autopsy is the only option for systemic effect analysis, which can bias data by showing only a worst-case scenario view of the pathogen’s etiology. Other study methods, such as PCR, whole genome sequencing, and immunoassays, only capture data at the point of sample collection and can only look for known processes or markers^6,8^.

MI provides the ability to obtain longitudinal whole-body data, allowing pathological changes to be monitored and detected in unexpected body regions^9^. For example, COVID-19 was initially considered a respiratory illness; however, it is now clearly identified to be multi-systemic, with pathology extending far beyond the respiratory system^10^. MI has great potential in pandemic situations, allowing rapid full-body assessments that could reveal the extent of pathology. Additionally, MI has a time advantage over other methods of studying viral pathology because probes are designed to measure specific processes or molecular markers—extending the range of monitoring beyond tissue-level changes to processes and biochemistry. MI probes allow in vivo detection of changes on the cellular or molecular level, accelerating the detection of both viral and viral-mediated pathology and opening the possibility to predict and prevent pathologic outcomes.

### Molecular imaging

The field of MI has seen rapid growth in the past decades. This progress, combined with other advancements in health sciences and personalized medicine, has led to the use of MI as a valuable research and clinical tool^1^. As mentioned previously, MI is generally non-invasive, useful for longitudinal studies in a single subject, which reduces variability and often enables whole-body imaging. Molecular imaging enables the study of biological processes, not just anatomy.

Anatomical imaging can look for larger-scale changes produced by malignancies or diseases, such as ground glass opacities in the lungs of pneumonia patients that can be seen on computed tomography (CT)^11^. CT and magnetic resonance imaging (MRI) are the most common anatomical imaging modalities, though X-rays and ultrasound are also possible^12^. In the case of COVID-19, ultrasound has been used to look at the structure of the lungs, kidneys, and heart and look for possible virally mediated changes^13^. Due to the focus of ultrasound on vasculature studies on anatomical imaging, it will not be discussed further in this work.

Anatomical imaging is also commonly combined with other MI modalities to provide a scaffold on which to overlay probe signals and provide structural clarity. The combination of anatomical imaging and functional MI allows for the visualization and localization of early changes in cellular physiology that can be detected before any anatomical changes or physical symptoms are present. One such example is metabolic imaging probes, like ^18^F-FDG, which are used to monitor cellular glucose uptake. MI also includes probes designed to target specific processes or molecular markers—providing a wealth of biochemical data. MI is commonly divided into nuclear, optical, and magnetic resonance (MR).

### Types of molecular imaging

#### Nuclear imaging

Positron emission tomography (PET) and single-photon emission computerized tomography (SPECT) are two of the most common nuclear imaging modalities^14^. Often, these are coupled with anatomical scans such as CT or MRI to correlate probe signals with anatomical structures and regions.

**Fig. 1:**
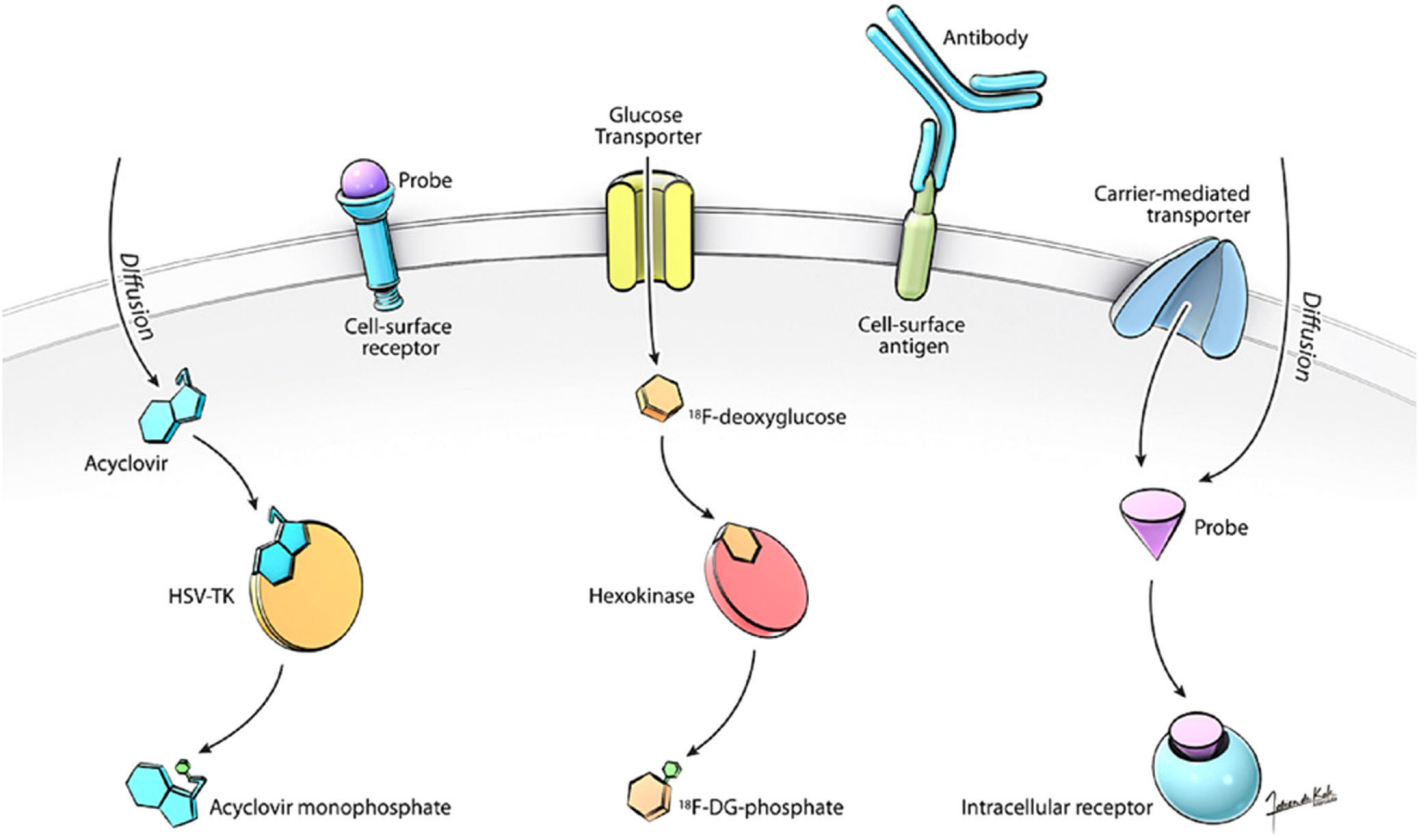
Examples of types of nuclear imaging probes based on different cellular targets. Figure from Bray et al. (2010), made by Fabien De Kok-Mercado. Image shows types of probes that can be used in SPECT and PET, and in some cases optical imaging. Probes may be designed to target and bind cell surface receptors via ligand mimicry or can be antibodies that recognize cell surface antigens. Other probes require internalization, with the specific intracellular target determining the mechanism of probe entry^16^.

Nuclear imaging uses probes comprised of radionuclides that interact with biological targets by binding and coupling to ligands (see examples in the section on probes used in virus MI)^15^. The tracers used are diverse, with different radionuclides, routes of administration, and tissue affinities available (Fig. 1)^16^. The choice of PET vs. SPECT imaging depends on the radiotracer of interest, its specificity, and the required spatial resolution to visualize the areas of interest. There are several factors when deciding what radiotracer to use, but the imaging target and half-life of the tracer are critical. Other factors to consider include the radiochemistry, pharmacokinetics of the tracer and its interaction with metabolism, whether repeated or single imaging is desired, and whether direct or indirect labeling will be used. Half-lives differ substantially between radioisotopes, with times ranging from approximately 2 minutes (^15^O) to several days (^89^Zr)^17,18^.

An ideal half-life depends on the uptake time needed for the imaging target (tissue or process of interest) and the ability to use the compound before decay, with additional considerations for repeated imaging and total radiation dose. Some advantages of nuclear imaging are the high detection sensitivity, range of potential probes and depth of penetration^19,20^. One of the disadvantages of these techniques is the reduced spatial resolution and the use of ionizing radiation.

PET imaging uses positron-emitting isotopes that collide with electrons to emit detectable annihilation photons^21^. A 3D image can then be digitally reconstructed from the data. PET tracers have been conventionally used to look for changes in metabolism or blood flow, but have many other applications, some of which are explored in this review^15^. PET is commonly used as the standard of care for oncological studies, where it can be used to detect changes in tumors or metastases early on^20^. However, the availability of PET radioisotopes, particularly those requiring a cyclotron for production, can be a drawback, as well as the short time frame for necessary radiochemistry to be performed on short half-life tracers. This can be both facility- and cost-prohibitive, making PET one of the more costly nuclear imaging modalities^22^. Traditional PET permits only one tracer to be detected at a time as the annihilation photon has an energy of 511 keV regardless of the radioisotope from which it originated^21^. However, recent work by a number of groups has demonstrated a novel method for multiplexed PET (mPET), which allows for the use of two isotopes simultaneously through the use of one positron emitting and one positron and gamma emitting isotope^23^. The two signals can then be resolved using the detection of the number of coincident events that are produced by each isotope. More information on mPET is available from Pratt et al.^23^.

SPECT also relies on the detection of photons to reconstruct images. In this case, probes undergo gamma decay, releasing photons that can be detected by gamma cameras^24^. Probes for SPECT imaging usually consist of a selected radioisotope conjugated to a biological ligand, allowing the modality to be modified for specific biological targets. There are several possible isotopes for SPECT imaging, with the most common clinical choices being ^99m^Technetium, ^123^Iodine, and ^201^Thallium^24^. One advantage of SPECT is the potential for multi-radioisotope imaging to simultaneously look at multiple biological targets. As SPECT is not limited to 511 keV photons, gamma, and X-rays of differing energies can more easily be distinguished in a single scan, so long as they are sufficiently separable^25–27^. While it is theoretically possible to image several isotopes at once, there is a practical limit to the computational ability to correct the crosstalk that results from multiple energy windows. For this reason, dual isotope imaging is most often reported, with some researchers having had success with triple isotope imaging^25–27^. Lukas et al.^25^ and Prieto et al.^26^ discuss the use of two different SPECT scanners for multi-isotope imaging and go into more detail about image acquisition and crosstalk correction. It should be noted that both PET and SPECT remain primarily single isotopes for most scans.

There are also differences in spatial resolution between the two imaging modalities, with PET having higher resolution in clinical scans, and SPECT in preclinical imaging^28–30^. Furthermore, PET has increased sensitivity compared to SPECT, due in part to the collimators used in SPECT resulting in loss of incident radiation^31^. The differences in sensitivity mean that PET and SPECT are often used for different applications, with PET being favored due to its fast scan time, and SPECT being useful in cases where long reaction time is needed^31^.

#### Optical imaging

Optical imaging is generally subdivided into bioluminescence imaging (BLI) and fluorescence imaging (FLI). Optical imaging has some advantages over nuclear imaging: it is inexpensive and does not require ionizing radiation^22^. However, due to the nature of the optical spectrum, this technology has diminished tissue penetration. This technique is better when looking at structures close to the skin or for use in smaller animals, where full depth may be achievable, though it can also be used in endoscopy or intraoperatively, as discussed in the context of brain tumor resection by Bin-Alamer et al.^32^. For an image to be created, the emitted signal must be stronger than the background noise that leaks through the filters, with that leakage determining how sensitive imaging can be^33^. Due to the ease of distinguishing different optical wavelengths, optical imaging can use multiple probes conjugated with different light-emitting compounds to image several targets simultaneously. This could prove highly advantageous for imaging viral pathology by labeling different immune cell populations involved in inflammation due to the heterogeneity of immune responses^34^.

Bioluminescence is a form of optical imaging used frequently in preclinical research but has yet to see widespread clinical implementation due to the need for transgenic cells^35^. Bioluminescence imaging (BLI) has been used in research settings to study inflammation, lung pathologies, and tumor dynamics^36^. Bioluminescence refers to light that is produced through a biological enzymatic reaction^36^. These enzymes, such as luciferase, oxidize the injected substrate, i.e. luciferin, producing bioluminescence. The enzymes can be activated through additional injection of the substrate to emit light repeatedly. This allows for non-invasive monitoring of disease processes in a single animal^36^. Transgenic animals expressing luciferase on critical cells or cell lines are most commonly used. Signal is produced by the injection of the substrate luciferin, which produces bioluminescence when oxidized^35^. Newer probes use what is known as caged luciferin, which prevents it from being a luciferase substrate until it is “released”^35^. This allows bioluminescent monitoring of metabolic processes, as the cage can be designed to be opened by desired reactions^35^.

Fluorescence imaging (FLI) works similarly to BLI; however, the light emission source differs. In the case of fluorescence, the emission is not produced by an enzymatic reaction; instead, each fluorophore has unique excitation and emission spectra. The fluorophore absorbs a photon at a specific wavelength and emits a lower energy photon shortly after^37^. The shift to a lower energy emitted photon is known as a Stokes shift. The larger the Stokes shift, the more easily excitation and emission photons can be distinguished^37^. Critically, a fluorophore can be excited again once it has returned to a ground state, allowing a single molecule to emit many photons in a short period of time^33^. This is in contrast to nuclear imaging, where the emission of a photon is linked to a finite decay process.

Near-infrared (NIR) imaging can improve the depth of tissue penetration that can be obtained compared to more traditional methods of optical imaging^38^. NIR imaging involves the administration of a fluorescent or bioluminescent agent that is excited above 780 nm^33,39^. This agent may be conjugated to a biological probe for targeted imaging, or it can be non-specific and used for applications such as angiography or lymphatic function^33^.

#### Magnetic resonance imaging (MRI)

Magnetic resonance imaging (MRI) requires a strong magnetic field to generate a net nuclear magnetization that can be manipulated by radiofrequency pulses to generate a signal that can be used to make an image^40^. The evolution over time of that signal is governed by so-called relaxation times—e.g., T_1_ and T_2_—which are dependent on the molecular and physical structure of the tissues^41^. The inherent contrast in MRI is the contrast between tissues without adding contrast agents or imaging probes—it is determined by the T_1_ and T_2_ values and the composition and density of the materials being imaged^42^. Many biological processes will cause changes in T_1_ and T_2_ of specific tissues, leading to MRI images being described as T_1_ and T_2_ “weighted”^43^. This work will not discuss the inherent contrast of MRI in more detail, as it will focus on the use of contrast agents or probes to modify relaxation parameters. Please refer to Deoni, 2020, for more information on relaxation^43^.

Different materials can be used as MRI probes due to their impact on relaxation times, including metals such as gadolinium or iron. Materials can be classified as non-magnetic, paramagnetic, or superparamagnetic^40^. Paramagnetic materials have unpaired electrons and lead to small increases in magnetic field strength but are not themselves magnetic^40^. Superparamagnetic materials cause significant increases in the magnetic field strength and are magnetic, meaning that image distortion from them must be accounted for. Usually, MRI probes are para- or superparamagnetic, though others (i.e., amides) for chemical exchange saturation transfer (CEST) probes are also available^5^.

MRI has several advantages relating to the quality of images that can be obtained and the fact that high-resolution anatomical and physiological data can be obtained simultaneously regardless of the use of probes. Furthermore, the lack of ionizing radiation eliminates possible harmful side effects linked to nuclear imaging. However, MRI scan times are longer than other imaging modalities, there is generally reduced sensitivity compared to nuclear and optical imaging, and the cost to set up and operate the scanner can be prohibitive^22^.

## Probes currently used in virus MI

Molecular imaging for the study of viruses is in its infancy. Studies have used two main techniques to image viruses or viral processes: direct labeling of viral particles and imaging biological processes to study host-pathogen interactions^44^. These processes can be monitored using small molecules, antibodies or antibody fragments, metabolic probes, which allow for direct measurement of metabolic features, and probes that use luciferase and quantum dots to enable high-fidelity imaging of inflammation. The many options result in numerous opportunities to measure different aspects of viral life cycles and pathogenesis. This section will discuss representative examples of different probes that have been used for viral imaging, with more examples included in Table 1.

**Table 1:** Additional examples of molecular imaging probes used to image viruses or processes resulting from viral infection. The table describes the general mechanisms of action of the probe, as well as key findings from studies that used it

Modality	Probe	Target	Virus	Mechanism of action
PET/CT	[^18^F]F-AraG,	Activated T cells	HIV	Studied in combination with ^89^ZR-VRC01. Found that there was a correlation between tissues with increased ^89^ZR-VRC01 and [^18^F]F-AraG uptake in HIV-positive patients. In particular, [^18^F]F-AraG was increased in lymphatic tissues and the bone marrow^134^.
PET/CT	^89^Zr-Df-Crefmirlimab	CD8 + T cells	COVID-19	Crefmirlimab (or IAB22M2C) is a minibody that binds to CD8 + T cells. This study used total body PET to look at CD8 + T-cell distribution dynamics in convalescent COVID-19 patients. They found that the ratios of CD8 + T cells in tissue:blood increased in COVID-19 patients compared to the control group, with this trend increasing over time^135^. A minibody was used over a full-sized antibody due to the small size, which has faster serum clearance^135^.
PET/CT	[^64^ Cu]NODAGA-CG34	CMKLR1— lung inflammation and injury	COVID-19, ARDS	CMKLR1 is involved in the recruitment of various immune cell populations to tissues. In the lungs specifically, this receptor has been shown to promote inflammation from irritants, infection, and injury, and in the case of COVID-19, it has been linked to macrophage recruitment leading to acute respiratory distress syndrome (ARDS). They found that CMKLR1 expression was 3X higher in COVID-19 patients compared to controls. In imaging of lung injuries, the authors report that regions of increased tracer uptake correlated to ground glass opacities seen on CT. Furthermore, treatment with dexamethasone in mice with LPS-induced lung damage resulted in decreased tracer uptake, indicating its specificity for lung inflammation^136^.
PET/MRI	[^18^F]FEPPA	TSPO	Zika	FEPPA is a ligand for TSPO that can be used as a PET marker of neuroinflammation. A study on non-human primates found that [^18^F]FEPPA could identify CD68+ myeloid cells in the brain in an allograft model^137^. A murine study on ZIKA found that of all immune cells present in the brain, only myeloid lineages showed increased TSPO compared to pre-infection. In the case of microglia specifically, there was a strong correlation between TSPO expression and [^18^F]FEPPA uptake^76^.
PET/CT	[18 F]DPA-714	TSPO	COVID-19	TSPO is distributed throughout the body, thereby serving as a marker of inflammation in multiple regions. Lung lesions caused by COVID-19 infection are difficult to visualize using anatomical imaging unless they are severe. Therefore, using [18F]DPA-714 is promising in identifying lung lesions based on increased immune cell migration into damaged regions. It was noted in preliminary non-human primate studies that increased TSPO signal correlated to increased dendritic cell levels even in tissues showing no anatomical signs of lesions^138^. Another group used [18 F]DPA-714 to look at neuroinflammation in long COVID patients. They found that those experiencing symptoms had substantially increased tracer uptake in the brain compared to matched healthy controls^139^.
PET/MRI	[^11^C]PBR28	TSPO	COVID-19	Glial cells can become activated in response to immune signals, but sustained activation can lead to disequilibrium between various cells of the brain. Persistent glial activation has been suggested as a possible component of neurological and vascular changes observed in long COVID. [^11^C]PBR28 is a ligand for TSPO, previously discussed as a marker of neuroinflammation. Using [^11^C]PBR28, large regions of increased uptake were observed in several brain regions, and the intensity of PET uptake in the brain correlated to markers of vasculature changes^140^.
PET/CT	^18^F-α_v_β_6_-BP	α_v_β_6_	COVID-19	^18^F-α_v_β_6_-BP is a probe designed to bind to the integrin α_v_β_6_ α_v_β_6_ is normally expressed at very low levels in healthy tissues; however, it is increased during tissue remodeling and fibrosis. This probe was tested on one patient approximately two months after COVID-19 recovery. Preliminary data found a good correlation between regions of increased ^18^F-α_v_β_6_-BP and CT findings (ex: GGOs) of COVID-19-mediated lung damage^141^.
PET/CT	^68^Ga-grazytracer	Granzyme B	Cardiac inflammation and myocardial infarction	Myocardial infarction (MI) has high morbidity and mortality rates, and can be triggered and linked to several different viral infections^64^. Recently, CD8 + T cells that release granzyme B have been shown to contribute to myocardial inflammation, with increased levels of these cells occurring in MI. The tracer ^68^Ga-grazytracer is a peptide-based tracer that selectively inhibits granzyme B via binding^142^. In rat models of MI, it was found that the tracer had the most accumulation in MI subjects compared to controls. This uptake corresponded to CD8 + . T cell and granzyme B levels were seen using immunohistochemistry^143^.
BLI/FLI	nLuc and ZsGreen1 [ZsG]	rMA-EBOV	Ebola	Using reporter nLuc or ZsG coupled to rMA-EBOV (a recombinant reporter virus) was sufficient for tracking the Ebola virus in vivo and ex vivo. While single-reporter recombinant viruses are available, the use of a dual-reporter system had comparable viral attenuation. As FLI has reduced depth penetration compared to BLI, dual imaging can compensate for this. Conversely, as BLI imaging modalities are largely limited to small animals, including an FLI reporter can also make the probe applicable to other species (despite the need for ex vivo analysis for deeper tissues)^144^.

### Nuclear imaging

Please note that all nomenclature for nuclear imaging probes follows the naming conventions from the original papers referenced to ensure consistency.

#### Monoclonal antibodies for direct viral imaging

Monoclonal antibodies (MAbs) are raised against a single antigen epitope, making them highly specific for their target. Currently, MAbs have been used in the treatment of several diseases, including but not limited to various cancers, autoimmune conditions, and infectious disease therapy. MAbs work through interaction with their target, which can have a wide range of effects, from interfering with malignant cell growth to immune-lead destruction of target cells. Due to the specific nature of MAbs, they have also been used as probes in molecular imaging, targeting specific epitopes of interest in vivo. An example of one such probe is ^89^Zirconium-VRC01, which was designed to target the human immunodeficiency virus (HIV)^45,46^.

The treatment of HIV has made astounding progress in the last decades with the use of antiretroviral therapy (ART); however, lifelong treatment is usually required^46^. One challenge of functionally eradicating HIV from patients is the ability of the virus to survive in infected cell reservoirs, complicating ART dosing and prediction of disease progression^46^. Notably, the gut-associated lymphoid tissue (GALT) of the digestive tract is a known reservoir of HIV and is a barrier to complete viral eradication, even in those on ART^47^. However, other tissues and organs can serve as reservoirs, making the use of MI a promising non-invasive method of evaluating the presence of HIV-1 throughout the body.

^89^Zr-VRC01 binds to a highly conserved epitope of the HIV surface glycoprotein gp120, analogous to how the virus would bind its target CD4 + T cells^45^. This interaction results in a cascade of conformation changes that eventually results in viral fusion of HIV to target cells^45^. VRC01 was initially studied regarding its potential to lead to virus neutralization. Studies found that while mutations to the gp120 protein of HIV could reduce VRC01 binding affinity, viral escape did not occur^45^. These findings support the use of ^89^Zr-VRC01 as an imaging probe that can withstand (to an extent) mutations to its target virus. However, in the long-term, it is likely that with rapidly mutating viruses such as HIV-1, a sustainable and efficient approach to probe design would have to be individualized to epitopes to ensure continued probe binding.

**Fig. 2:**
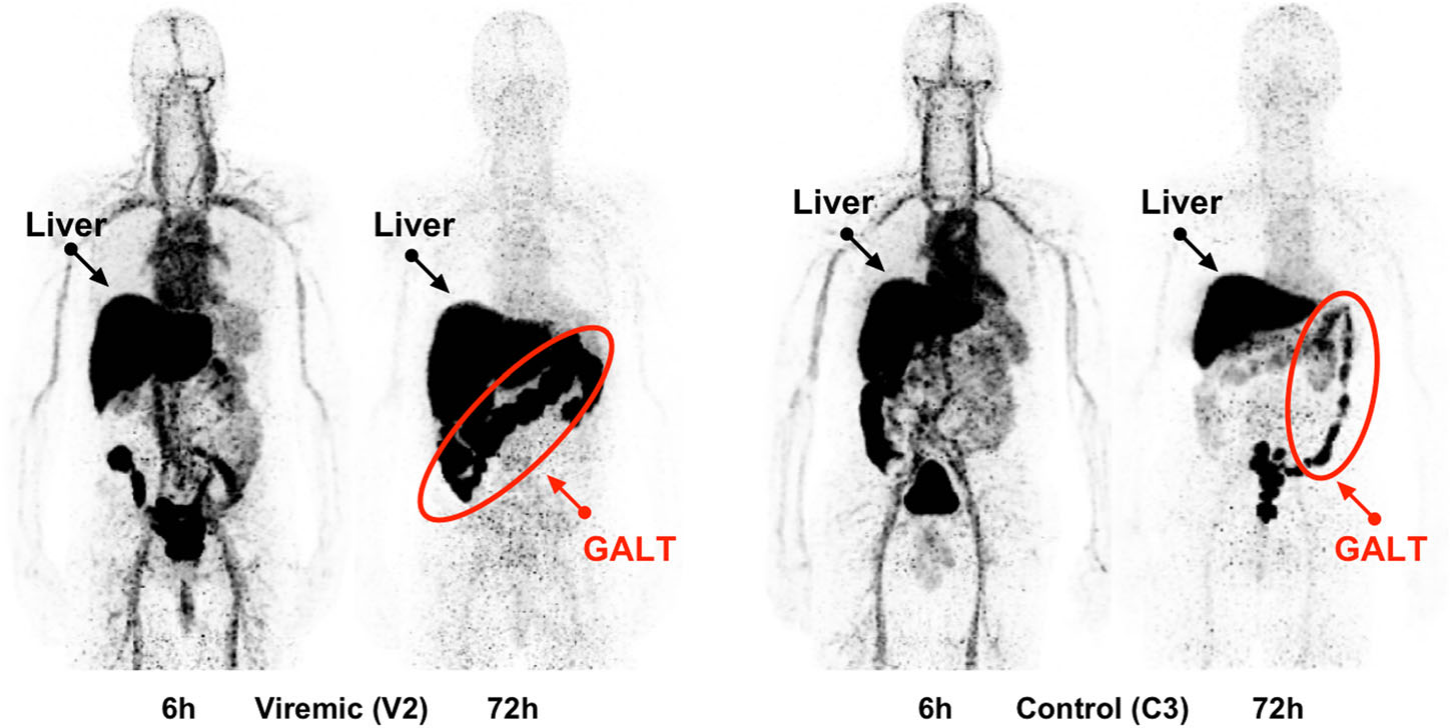
Using ^89^Zr-VRC01 to monitor HIV infection. Image taken from Beckford-Vera et al. (2022). Image shows uptake of the tracer ^89^Zr-VRC01 in a viremic HIV-positive volunteer compared to uninfected control. Images were acquired 6 and 72 h post-probe administration using MRI for anatomical background and PET for probe detection. Dark regions on the images show areas of increased ^89^Zr-VRC01 uptake^46^. Liver and GALT labels were added. GALT is circled in red. The Creative Commons license for the figure can be found at http://creativecommons.org/licenses/by/4.0/.Be.

PET/MRI imaging found that accumulation of ^89^Zr-VRC01 was increased in those with active viremia and those on ART compared to uninfected controls (Fig. 2)^46^. This trend was particularly apparent for the GALT but was also noted in the digestive system and some bone marrow (not shown in the image)^46^. Due to the link between the GALT and viral persistence, the increased uptake of the ^89^Zr-VRC01 in the gut is promising to monitor HIV reservoirs in ART and non-ART patients. This probe also benefits from using ^89^Zr as the radionuclide due to its long half-life, which allows for probe detection over several days^46^.

#### Antibody fragments for imaging T-cell recruitment

Nanobodies are fragments of antibodies that are made up of a single variable domain. This allows them to have epitope specificity while being smaller in size. Using nanobodies as imaging probes rather than full-sized antibodies has several advantages. Namely, the reduced size of nanobodies extends the size range of possible targets, including those too small to be targeted by MAbs^48^. Furthermore, the small size provides better clearance and dispersion through the body^48^. However, like all antibodies and antibody fragments, nanobodies have the potential to be immunogenic^48^, though the risk is low due to the high sequence similarity between the camelid VHH used for nanobodies and humans^49,50^. The nanobodies are specific, easily chemically adaptable, and can be paired with a variety of radionuclides depending on the desired half-life. One example of an imaging probe based on a nanobody is ^89^Zr-VHH-X118, which has been used to investigate CD8 + T-cell migration in murine models of Influenza A virus (IAV)^50^. While this study was performed in mice, there are several examples of nanobodies being used clinically to monitor CD8 + T-cell responses^51–53^.

Influenza A virus (IAV) is implicated in both seasonal outbreaks of influenza and epidemic or pandemic viruses^48^. IAV can infect the upper and lower respiratory tract, with infection usually triggering an inflammatory immune response^48^. Part of the inflammatory response to IAV is the recruitment of various immune cells to the site of infection, such as CD8+ cytotoxic T lymphocytes (CTLs). CTLs specific for IAV are suspected to lead to airway damage by secreting proinflammatory mediators, including granzyme, IFNγ, and TNF-α, amongst others^48^. Therefore, understanding the patterns of CD8 + T-cell recruitment during IAV infection is beneficial for understanding the disease dynamics.

**Fig. 3:**
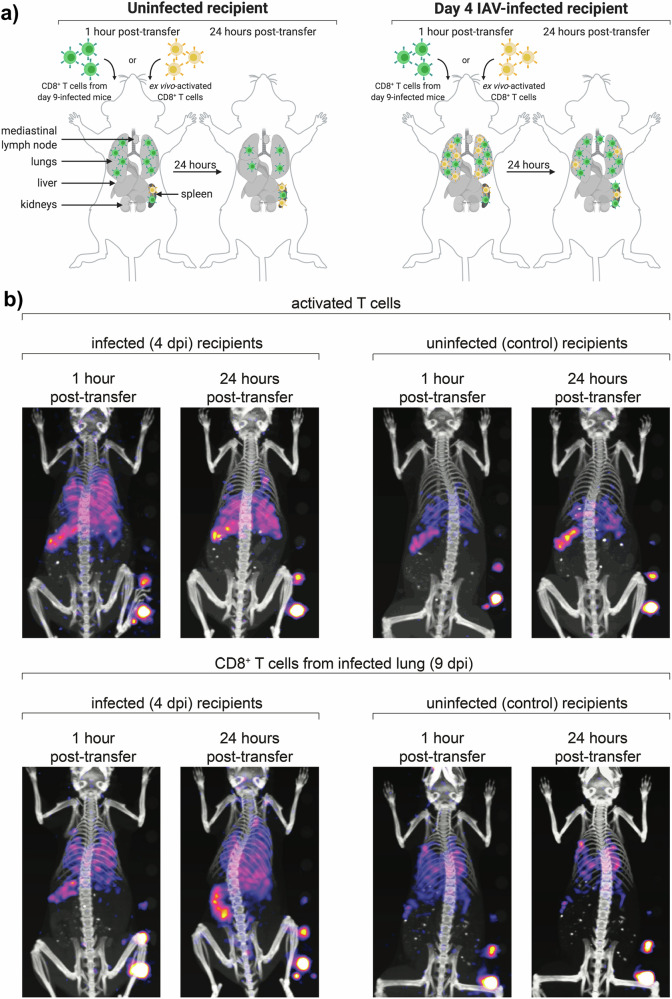
Monitoring CD8 + T-cell recruitment in a murine model of IAV. Image from Rothlauf et al.^48^. **a** Image shows a schematic of CD8 + T-cell adoptive transfer (Green) from mice infected with IAV for 9 days to either uninfected controls or mice infected with IAV for 4 days. Yellow CD8 + T cells represent cells that were activated ex vivo and are not specific towards IAV antigens. 1 h post transfer showed recruitment of adoptively transferred cells to the lungs and spleen in both cohorts, with increased pulmonary retention at 24 h in the infected mice. Very few ex vivo activated cells were recruited to the spleens and lungs in uninfected controls. While more ex vivo cells were recruited in infected mice, they were not retained as highly as the adoptively transferred cells. **b** MRI/PET images of representative mice given either ex vivo activated CD8 + T cells or adoptively transferred CD8 + T cells from day 9 infection^48^.

Studies using ^89^Zr-VHH-X118 initially found that there was poor resolution in the spleen, likely due to the high concentrations of CD8 + T cells known to reside there. This was solved through the use of a pegylated version of the nanobody, which resulted in good resolution in the spleen and secondary lymphoid organs and reduced non-specific probe uptake in the kidneys^48,54^. ^89^Zr-VHH-X118 has also been used to label CD8 + T cells in adoptive transfer experiments, with recruitment of the transferred cells being observed in the lymph nodes and the lungs. Recruitment changes were correlated with disease progression, with early infections showing a diffuse ^89^Zr signal while later infections showed concentration in the lungs (Fig. 3)^48^. Due to the use of ^89^Zr, the signal from the probe could be detected for up to 4 days post-injection, allowing for multiple imaging sessions within that time to track real-time changes in CD8 + T-cell migration.

Other studies have also used antibody fragments to study the presence of CD4 + T cells in non-human primates (NHPs) in the context of Simian immunodeficiency virus (SIV). In this study, the probe used was an antibody fragment probe CD4R1-F(ab′)_2_ mAb that binds to domain 1 of the receptor of CD4 + T cells^55^. This probe was labeled with ^89^Zr, allowing for up to 6 days of imaging. This probe was able to successfully distinguish CD4 + T cells in lymphoid tissues, and differences between immunocompetent and immunocompromised NHPs were noted^55^.

#### DNA salvage probes to monitor activated T cells

[^18^F]F-AraG is an arabinosyl guanine analog, which itself is an analog of deoxyguanosine^56^. T cells change their metabolism when they become activated, using substrates different from those of their non-activated counterparts^57^. Purines and pyrimidines are critical DNA building blocks that activated T cells need to maintain their proliferation rate. The proliferating cell can make these molecules, but they also can be salvaged through pathways that break down ribonucleoside diphosphate and deoxynucleosides, such as deoxyguanosine^58^. [^18^F]F-AraG probes take advantage of these metabolic alterations to provide more specific activated T-cell identification, as [^18^F]F-AraG can be metabolized by the salvage pathway enzyme deoxyguanosine kinase (dGK)^57–59^. In activated T cells [^18^F]F-AraG is taken up as a substrate for the dGK pathway; instead of being broken down, it accumulates within these activated cells, allowing it to be detected with PET^57^.

[^18^F]F-AraG has primarily been used in oncological imaging. In tumors, the accumulation of [^18^F]F-AraG has been found to correspond well to the location of CD8 + T cells, validating its use for tracking proinflammatory immune proliferation^56^. One possible application of [^18^F]F-AraG probes to study viral pathology is to monitor the activation of T cells in response to infection. Much like this is done in cancer models, activated T cells can be assessed to determine where they localize, giving an indirect idea of where viral foci could be located in addition to monitoring changes in T-cell migration and infiltration, the probe would be useful in studying the T-cell response to anti-viral therapies. Finally, the probe would also be beneficial in testing T-cell-based vaccines, with increased uptake indicating increased levels of activated T cells.

An example of the use of this probe for imaging viruses can be found in a study by Peluso et al. (2023), which describes the use of [^18^F]F-AraG PET/CT imaging to look at T-cell activation in COVID-19^60^. The authors imaged patients up to two and a half years following COVID-19 infection, some with symptoms of long COVID and some without. This was done as clinical data on T-cell changes, and COVID-19 was limited to small cohorts or samples taken during acute infection. By using [^18^F]F-AraG, the authors observed that there were increases in the concentration of the probe present throughout the body in those infected with COVID-19 compared to uninfected controls from before the pandemic^60^. Furthermore, they found that increased [^18^F]F-AraG concentration in the spinal cord and gut wall correlated with the likelihood of experiencing long-term COVID symptoms^60^.

#### Peptide probes to monitor CD8 + T-cell effector function

Peptide-based probes are a type of small molecule probe that demonstrate good specificity (albeit less than mABs), structural flexibility, and relatively low immunogenicity profiles^61^. These probes can be subdivided into three classes based on their mechanism of action and their bonds to detection moieties^61^. There is a wide array of possible peptide-based probes that have been tested for use in molecular imaging, and which can be conjugated with different signaling molecules for different types of imaging^62^. One example is the use of a peptide against granzyme B. Classically, CD8 + T-cell effector functions are linked to CTLs, which produce different cytokines and effector molecules such as granzyme B, IFNγ, and TNF-α that can lead to the death of the target cell.

Using a restricted interaction peptide (RIP) specific for granzyme B, PET/CT imaging was able to detect regions of increased granzyme B-driven proteolysis. This was studied in response to infection with H1N1 influenza or in acute bacterial infections. They tested both wild-type mice and mice that did not produce granzyme B with the [^64^Cu]Cu-GRIP B probe. They found that in wild-type mice infected with H1N1, there was significantly increased signal detection compared to mice that received a mock infection or were knockouts for granzyme B expression^63^. This type of probe is promising to study the effector functions of CD8 + T cells, rather than just if they are present or not in a particular tissue. Peptide-based granzyme B probes have also been used to study myocardial infarction (MI), a condition with high morbidity and mortality, and which several viral infections are known triggers for (see Table 1)^64^.

#### TSPO imaging of neuroinflammation

DPA-714 is a ligand for the translocator protein 18 kDa (TSPO), a protein located on the outer membrane of mitochondria, linked to several cellular processes such as apoptosis, immune modulation, cell proliferation control, and mitochondrial bioenergetics^65,66^. It is upregulated in a variety of disease conditions but notably is linked to neuroinflammation, being seen at increased levels in neurological disease^65,66^. Furthermore, it has been seen that microglia are the predominant cell expressing TSPO in CNS diseases, correlating to the known role of these cells as markers for neurological conditions^66^. TSPO has also been suggested as a biomarker of peripheral tissue inflammation, showing high expression on macrophages during sterile inflammation^67^. The use of DPA-714 PET probes targeting TSPO has been suggested as a method to monitor macrophage migration in various organs. While the exact mechanism of DPA-714 probe entry into cells is unclear, a kinetics study showed highly specific binding to TSPO, indicating efficient cell entry^68^.

An ^18^F-DPA-714 probe has been tested in the Ebola virus (EBOV) to look for neuroinflammation resulting from infection. EBOV is highly infectious and deadly and can infect several different cell types, leading to large viral dissemination^69^. Due to the nature of EBOV outbreaks, studies on its etiology are restricted to in vitro work or the use of post-mortem samples from animal models, making the use of MI to evaluate EBOV mediated pathology an attractive prospect^69^. Shah et al. used ^18^F-DPA-714 as a PET probe and various in vitro tests to investigate the etiology of EBOV^69^. This study found that the highest levels of ^18^F-DPA-714 probe binding were in macrophages and monocytes, keeping with the known expression of TSPO^69^. However, it was found that other cell types, including dendritic cells, T and B cells, and neutrophils, also showed ^18^F-DPA-714 binding, suggesting that probe levels would be markers of an overall inflammatory state rather than macrophages alone^69^. Critically, these cell types also expressed varying levels of TSPO, correlating to the PET findings^65^. Decreases in levels of CD3 + T cells and monocytes further correlated to reductions in ^18^F-DPA-714 binding^65^. The results of this preliminary study suggest the benefit of DPA-714 probes in assessing organ-level viral pathogenesis and for non-invasive monitoring of viral-induced inflammation, including in the brain. While the Shah et al. study looked specifically at EBOV, the ^18^F-DPA-714 probe has potential applications for PET imaging a wide range of pathogens due to the broad immune phenomenon being imaged. Table 1 describes the use of ^18^F-DPA-714 for imaging lung inflammation and gives examples of other TSPO imaging probes for the study of neuroinflammation.

#### Metabolic imaging of inflammation

^18^F-fludeoxyglucose (FDG) is a glucose-specific PET radiotracer that accumulates in tissues and cells with increased glucose metabolism. FDG is a glucose analog, meaning that it follows the same metabolic pathway as glucose—up to a point. Glucose is taken into cells and converted into glucose-6-phosphate (G6P) which can then enter glycolysis. ^18^F-FDG gets taken up by the cell and converted to FDG-6-phosphate; however, it cannot enter further metabolic pathways and becomes metabolically trapped^70^. While trapped in the cell, ^18^F-FDG undergoes radioactive decay, leading to an accumulation of signals within the cell that PET can detect. Tissues and cells with higher metabolic requirements will show increased ^18^F-FDG accumulation, a phenomenon known as the Warburg effect in cancer^70–72^. While ^18^F-FDG is specific for glucose, it is non-specific for biological processes, with any process that leads to increased glucose metabolism able to be detected but not distinguished from each other. This results in diverse imaging applications, including myocardial viability studies, brain metabolism research, oncological imaging, and more recently—imaging of inflammation and infection^73^.

In imaging of infection and inflammation, FDG uptake is increased due to immune cell recruitment to inflamed regions and the accompanying increase in glucose metabolism^74,75^. One example is a study looking for non-invasive biomarkers of Zika virus (ZIKV) disease progression, with the goal of using them to monitor therapeutic efficacy^76^. Imaging was done on a murine model of ZIKV given a lethal infection dose. The authors found that ^18^F-FDG uptake was increased in distinct body regions in diseased animals, with the increased uptake occurring most often in the spleen or other lymphatic tissues^76^. The increase of ^18^F-FDG in the spleen was in keeping with the severity of the illness and the levels of proinflammatory cytokines^76^. Earlier studies by the same group had described similar results for infection with dengue virus (DENV) and ^18^F-FDG uptake^77^. A recent study on COVID-19 out-patients used ^18^F-FDG imaging to research the role of the brain in symptoms of long COVID^78^. They found that there were brain regions in long COVID patients that displayed hypometabolism and that this was correlated with increased clinical symptoms during acute infection and more long-term neurological side effects^78^. Another group used ^18^F-FDG uptake to look for differences in myocardial tissue in COVID-19 patients based on vaccination status^79^. They found that vaccinated patients had increased myocardial FDG uptake compared to those who were unvaccinated. Those who reported pain at the vaccination site were found to be more likely to have increased myocardial uptake than those without vaccine side effects^79^. These studies highlight the potential of ^18^F-FDG to identify virally induced inflammation.

The use of ^18^F-FDG imaging for COVID-19 highlights the benefit of the non-specific nature of FDG imaging. That is, it can be used to investigate various inflammation-linked phenomena, such as vaccine-mediated inflammation as well as viral-induced tissue damage. However, the non-specificity of ^18^F-FDG also has some disadvantages, mainly stemming from the difficulty in directly attributing a cause to increased uptake^80^. As malignancies, infection, inflammation, and injury can all cause similar increased FDG uptake, stringent controls, and biological confirmations are required to pinpoint the cause. Different tissues also have different physiological uptake of FDG, and factors such as age, comorbidities, blood glucose levels, and even exercise before scanning can all impact imaging results^80^.

### Optical imaging

#### Monoclonal antibodies for optical imaging of viruses

There are several MAbs available that target viral epitopes of numerous different pathogens. As previously mentioned, these MAbs have the potential to be used as imaging probes—in this case, by conjugating them to fluorescent moieties for detection by optical imaging. An example of one such MAb-based probe is CR3022, which has activity against the spike protein of SARS-CoV-2. As the SARS-CoV-2 S protein is the primary mediator of viral entry into host cells, it is critical to the viral life cycle and, therefore, a suitable imaging target^81^.

This probe was designed to investigate the anatomical sites where SARS-CoV-2 caused infection due to the highly individualized nature of the disease^68^. The diverse symptoms produced by COVID-19 could be caused by direct viral infection at different sites or from downstream impacts of the virus, such as immune-mediated damage^82^. Several studies have used tissue analysis from animal studies or human clinical samples to look for the etiology of symptoms. However, these only provide single time point information, giving no indication of viral kinetics, past viral infection, or inflammation over time^82^. The use of the CR3022 antibody as the targeting moiety of a molecular probe allows for longitudinal imaging of a single subject. Notably, by correlating infected cells with symptoms of infection, a distinction between pathological causes is possible.

The signal from CR3022 would be present throughout the body, but as it targets the S protein, its accumulation will only occur in infected cells. CR3022 has been tested with several fluorescence signaling agents: Cy3, Cy5, and AF647^82^. Early studies performed fluorescent optical imaging of SARS-CoV-2 of infected rhesus-macaque tissue post-necropsy at pre-determined study time points, with data indicating good localization of the viral probe to sites of infection and viral replication^82^. The success of this study using CR3022 suggests that more research into the use of this probe as a marker for COVID-19 infection could provide insights into disease mechanisms. However, optical imaging has tissue penetration constraints due to signal attenuation in tissue. This is why only post-necropsy tissue analysis was possible for the rhesus-macaque study. In small animal preclinical models, the use of the CR3022 probe is more feasible due to the small size of the imaging subject. The authors of the preliminary study also suggest that CR3022 could be adapted for use as a nuclear imaging probe to obtain imaging data with better depth than FLI^82^. However, a noted risk of this probe is its possible neutralizing impact on the virus, possibly changing the kinetics of the infection^82^. Furthermore, due to the S protein mutation rate, the probe likely will need adaptation to new variants.

#### Optical protease-activable viral probes

While imaging of immune functions, cell recruitment, and inflammatory processes is valuable, the ability to directly image viruses is highly desirable. In particular, activatable probes that react to viral processes have the potential to give a quantitative and real-time metric of viral infection and spread. The SARS-CyCD probe is an optical imaging near-infrared (NIR) fluorescent probe that can be activated by the enzymatic activity of a SARS-CoV-2 specific protease (Mpro) that is critical in viral polypeptide cleavage and the viral lifecycle^83^. It is the first in vivo activatable probe for monitoring SARS-CoV-2 infection via optical imaging. The fluorescent moiety is caged in the resting probe, so the fluorescent signal is masked. When Mpro is present, CyCD is detached from the rest of the probe and activated to fluoresce^83^. This probe highlights the potential of specifically designed probes for viral enzymes to aid in MI, although the limits of tissue depth penetration with fluorescent imaging pose challenges for its use in humans. However, this probe would be beneficial in preclinical studies on small animals, where the required depth to visualize mouse lungs is significantly reduced compared to what would be required for humans.

#### Quantum dots for viral tracking

Quantum dots (QDs) are used as FLI imaging probes and are designed to emit in the first or second NIR window^84^. QDs are highly stable, showing less fluorescence signal quenching than organic fluorophores. They also have longer times in their excited state, making signal detection easier and reducing the effects of autofluorescence^84^. By coupling QDs to specific targeting moieties, they can be used to image specific molecules or cells of interest.

An example of the use of QDs for viral imaging is single virus tracking (QSVT)^85^. QVST works by tagging viral components with QDs, allowing for real-time viral tracking and providing insight into the mechanisms of viral infection on an individual cellular level. QDs are distinguishable from each other by size and fluorescence, making them suitable for multicomponent imaging and allowing several aspects of viruses to be monitored in tandem^85^. Though QSVT has currently been used only with microscopy, there is potential for it to be used for in vivo virus tracking. Preclinical studies using QDs to track breast and prostate cancer in vivo report that the fluorescence is detectable through up to 10 cm of tissue^86^. This suggests that QSVT could be further used in animal models for preclinical research and in clinical studies of tissues near the skin.

#### Luciferase-based probes for preclinical study of viruses

Luciferase (Luc)-based probes are commonly used for bioluminescent imaging (BLI) studies. While luciferase reporter genes are widely used in the in vitro study of viruses, in vivo imaging is still a newer use of the technology. A benefit of using luciferase reporter genes is that they can be expressed under virtually any promoter, making it an easily adaptable system^87^. Furthermore, due to a variety of available luciferases, potential issues of gene size for insertion can be largely mitigated so as not to disrupt target genes. Currently, there are two main strategies for using luciferase to study viruses: creating recombinant viruses that express luciferase and using transgenic mice that express it under the control of a relevant promoter^87^. As with other optical imaging technologies, using luciferase and BLI is more practical for preclinical models than clinical settings due to an average of 10x signal attenuation for every added centimeter of tissue^87^. This means that while BLI can be used clinically, it is best for imaging shallow tissues or for use in intraoperative settings. BLI has the additional disadvantage of requiring the transgenic modification for the addition of luciferase, which results in far more clinical hurdles.

Recombinant viruses expressing luciferase have been used to study several RNA and DNA viruses. Usually, the luciferase is inserted so that its expression is controlled by a viral rather than a host promoter to capture the dynamics of virus enzyme-mediated infection and replication. However, in some instances, such as with IAV, this strategy is complicated due to the lack of space in the genome for insertion without impacting the viral fitness^87^. In the case of human herpes viruses (HHVs), recombinant Luc viruses have been used to study latency and reactivation^87^. This was found by putting Luc under the control of the immediate early promoter, only reacting with luciferin when this promoter was active^87^. Studies on the role of viral genes are also possible, using different viral knockouts that express Luc only when replicating. In the case of DENV, studies into the neutralizing ability of a monoclonal antibody have been done using Luc-based BLI to observe viral fitness^88^.

Other strategies to study viruses include making transgenic mice that express luciferase, allowing unmodified viruses to be studied^87^. In the case of IAV, this approach can be highly beneficial as it avoids the issue of Luc insertion into the viral genome by using wild-type viruses. In one study, the production of type I interferon (IFN) was monitored via the induction of IFN-β using a luciferase under the control of the IFN-β promoter. This system revealed not only the constitutive expression of IFN-β in different tissues but also how it was activated in response to infection with IAV^89^. As a type I IFN responses are key anti-viral immune components and largely regulated by IFN-β production, this system is highly valuable for monitoring viral immunity. Furthermore, due to the versatility of Luc insertion under various promoters, this system can be adapted to study many aspects of viral infection beyond what has been tested in the literature.

## Current MI probes used in oncology with possible virological applications

Many of the myriad probes designed to measure cancer development and status, measure metabolic, structural, and cellular changes. Since these are fundamental processes, it opens the possibility of using these probes to study the features of infection and virus progression. Existing probes include those that look for cell death and those based on the use of viral enzymes as reporter genes. Metabolic probes are also often used in oncological MI, usually based on changes in energy requirements needed to sustain the rapid growth and proliferation of cancer cells. However, metabolic changes are also common in responses to infection (see Table 2 for a list of common metabolic imaging probes).

**Table 2:** Examples of metabolic probes used in different imaging modalities. The table describes the mechanism of action of each probe as well as the targets against which it has been used

Modality	Metabolic pathway	Probes	Target	Mechanism of action
PET	Glycolysis	^18^F(FDG)	Cells with increased glucose metabolism	^18^F(FDG) uptake is increased in energetically demanding cells—such as cancer cells^145^.
PET	Amino acid metabolism	*O*-(2-^18^F-fluoroethyl)-l-tyrosine	Gliomas	Low uptake in healthy tissue, increased in gliomas due to higher rates of protein synthesis and transport into the tissue^146,147^
MRI	Glycolysis	H[1-^13^C] pyruvate	Increased pyruvate to lactate metabolism	Rapid pyruvate to lactate conversion is a hallmark of aggressive cancer^148,149^.
Magnetic resonance spectroscopy (MRI)	Pentose phosphate pathway (PPP)	C^13^	Telomerase reverse transcriptase (TERT)	TERT is linked to metabolic reprogramming in various cancers, increasing glucose flux through the PPP^150^.
SPECT	Amino acid metabolism	_L_-3-^123^I-Iodo-*α*-methyltyrosine (^123^I-IMT) is	Increased protein metabolism	Gets taken into tumors due to increased amino acid transport but is not incorporated into proteins^151,152^.
SPECT	DNA synthesis	^131^I or ^124^I labeled 2-arabino-fluro-5-iodo-2-deoxyuridine (FIAU)	Proliferating tumors	Nucleosides (or analogs) are phosphorylated by thymidine kinases and then incorporated into nascent DNA. FIAU is a uracil analog and has been used to monitor the proliferation of tumors through its incorporation into DNA^152^. FIAU can be used with different modalities by changing the attached label—for example, ^14^C-FIAU is a PET probe^153^.
SPECT	Hypoxia	^99m^Tc-pano	Hypoxic tissues	Nitroimidazoles can act as a marker of hypoxia. This is because they are reduced to reactive products in conditions with little oxygen. These reactive products then become trapped in tissues^152^.
BLI	Glucose uptake	bioluminescent glucose uptake probe (BiGluc)	Regions of increased glucose uptake	Bioluminescent glucose probe that is taken up by cells with increased glucose needs. Used in animal models that express luciferase. A caged luciferin phosphine (CLP) is given to the animals, followed by glucose azide (GaZ) and imaging 24 h later. CLP and GaZ interaction releases luciferin that can react with luciferase to produce bioluminescence. As GaZ requires transport into the cell using glucose transporters (GLUT), luminescence will be produced proportionally to the amount of GaZ imported. Therefore, stronger bioluminescent signals will be detected in cells with increased GLUT activity to support their metabolic needs. BiGluc has been used to monitor reactions to GLUT blockers for therapeutic potential and has been considered a radioactivity-free alternative to ^18^F(FDG)^154^.
FLI	Lipid metabolism	boron-dipyrromethene (Bodipy) FL c16	Cells with increased fatty acid uptake	Palmitate analog to measure fatty acid (FFA) uptake. Increased uptake is linked to cancers such as triple-negative breast cancer and can show dysregulations of lipid metabolism. Increased FFA uptake is also linked to more aggressive tumors^155^.
NIR FLI	Lipid metabolism	AlexaFFA	Increased fatty acid metabolism	Consists of Alexa Fluor 647 conjugated to palmitic acid. Increased probe uptake in tissues with increased fatty acid metabolism^156^.

### Nuclear imaging

#### Nuclear Annexin-V probes to image apoptosis

Annexin-V is part of a larger annexin family that can bind to phospholipids^90^. The ubiquitously expressed annexin-V shows a high binding affinity for phosphatidylserine (PS), a known marker of apoptosis^90^. During apoptosis, cells undergo many changes, one of which is the redistribution of PS from the cytoplasmic side of the cellular membrane, to also be expressed on the cell surface^91^. This PS exposure outside of the cell allows annexin-V to bind, serving as a marker for apoptosis.

While PS exposure is mainly mediated by apoptosis, other cell death processes may also expose it^91^. This particularly applies to autophagy, though sustained high levels of Ca^2+^ can also result in PS exposure to a lower extent than in apoptosis^91^. While both of these causes of PS exposure are less frequent than apoptosis, they can lead to annexin-V binding. Several studies have found that cells under stress with higher Ca^2+^ show annexin-V binding in MI^91^. While this may act as a confounder in the search for apoptotic cells, it also could have enormous benefits to look for tissues that are highly damaged but not yet apoptotic^91^.

Several different approaches for MI probes using annexin-V have been tested. Most of these are radionuclide-based for SPECT imaging, but PET and optical imaging have also been used^91,92^. Early trials of imaging probes used annexin-V to look for chemotherapy and radiation therapy-mediated apoptosis to evaluate the efficacy of various treatments on tumors^91^. An early study published in 2002 found that a lack of annexin-V probe uptake during cancer treatment strongly correlated with tumor unresponsiveness^91,93^. While annexin-V-based probes for MI have been used frequently in oncology studies, there is a lack of literature describing their use for imaging viral infections. Based on the role of annexin-V and PS in identifying cell damage and apoptosis, these probes could be valuable tools for imaging infection. For example, viruses like HIV and IAV that are known to induce cell death can also mediate plasma membrane disruption, leading to PS exposure^94^. In fact, for HIV-1, there is data suggesting that exposed PS is critical for viral binding, infection, and replication^94^. In the case of HIV-1, PS also appears to have host protective roles, binding to budding viruses to retain them on the infected cell^94^. Due to the dual roles of PS regarding HIV-1 infection, annexin-V probes for imaging would give valuable information, although characterization of the role of PS would be required. Using annexin-V probes to study viruses would also provide information on levels of apoptosis occurring in the cells, giving insight into both damages from the infection and the immune response to it.

#### PET reporter genes and PET reporter probes for viral detection

PET reporter genes (PRG) work in tandem with pet reporter probes (PRP) to lead to probe accumulation in one of three ways: PRP phosphorylation by PRGs resulting in cell trapping, receptor-ligand binding between PRP and PRGs, or PRGs that mediate transport of PRP into the cell^95^. While many combinations of PGR and PRP exist (see Yaghoubi et al.^95^ and Gao et al.^96^ for more examples), this review will discuss a well-characterized system that uses the thymidine kinase (TK) of herpes simplex virus (HSV) as a PGR^97^.

The gene *HSV1-tk* and its mutant *HSV1-sr39tk* are commonly used with PET MI to examine cell trafficking in vivo^97^. In this case, the PRP is a substrate of HSV TK such as 9-[4-[^18^F]fluoro-3-(hydroxymethyl)butyl]guanine ([^18^F]FHBG)^97^. A significant benefit to this system is that [^18^F]FHBG is readily phosphorylated by the PRGs but poorly by mammalian TKs, making it specific to the virus. Therefore, only cells transfected with the PRG will process the PRP and show accumulation via trapping of it^97,98^. Initially, this probe system was developed as a suicide gene therapy for cancer treatment^97,98^. The idea behind this therapy is that tumor cells would be transfected with a viral or bacterial gene that codes for enzymes able to change a harmless prodrug into one that is toxic. Monitoring by radiolabelled PRPs before prodrug administration allowed transfected cells to be localized using PET^98^.

This elegant system of highly specific imaging is currently being underused. A literature search did not find reports of the use of *HSV1-tk* or *HSV1-sr39tk* and [^18^F]FHBG to monitor HSV1 directly, though it has been used previously for longitudinal imaging of T cells^96^. However, the mechanism of the system suggests that it would be ideal for tracking HSV-infected cells in vivo. Without *HSV1-tk* or *HSV1-sr39tk* transfection of cells, only those infected with the virus would have the TK required to accumulate the PRP. In the same way that transfected cells can be localized, infected cells should be apparent. Furthermore, as transfection of cells would not be needed, this could have clinical applications in the study and diagnosis of viral infections. A disadvantage of [^18^F]FHBG is that it does not cross the blood-brain barrier, meaning it could not be used for studies on viral infection in the brain^96^.

### MRI

#### Superparamagnetic iron oxide (SPIO) for immune cell tracking

SPIO is a contrast agent (probe) that is used in MRI. Its mechanism of action is simple, with cellular uptake resulting in hypointense contrast on *T*_2_^*^ weighted scans^99^. Clinically, SPIO is often delivered intravenously for imaging of liver lesions and liver cancers. There are different options for labeling cells of interest with SPIO. One route is via injection to take advantage of the spontaneous uptake of SPIO into phagocytic cells. In research settings, SPIO cell labeling can also be done ex vivo using cultured cells. Cell populations of interest can be taken from patients or research models and cultured with SPIO to allow for uptake before being adoptively transferred back into the organism^100,101^. SPIO-enhanced MRI has been used to monitor the impact of immunotherapies, particularly cancer vaccines, and to track immune cells^102,103^.

One option for tracking viral pathogenesis is based on the preferential SPIO uptake by reticuloendothelial system cells (RES). Both bone marrow and circulating phagocytic cells, primarily macrophages, take up injected SPIO. While the uptake is not specific to macrophages, this method indirectly examines the accumulation of phagocytes in specific body regions in response to infection. For example, in SARS-CoV-2 disease, macrophages are key components of early viral antigen recognition and immune presentation^104^. SPIO could also be used to label various immune cell populations ex vivo or to label antibodies responsive to different viral components.

#### Chemical exchange saturation transfer (CEST) probes for MRI macromolecule tracking

CEST is an MRI technique based on the exchange of protons between solutes and bulk water molecules^105^. A significant benefit of CEST is that it increases the detection limit of markers that normally could not be detected by MRI or would necessitate toxic levels of binding probes^105,106^. Currently, CEST is used to image either contrast agents or endogenous molecules of clinical interest, with the most common use being the imaging of amide protons in the detection of cancer^106^. Other studies have reported using CEST to image glucose, glycogen, glutamate, enzyme activity, and lactate, amongst other markers^107–112^.

As discussed previously, increased rates of glycolysis metabolism and glucose uptake are characteristic of both tumor cells and inflammatory immune processes. GlucoCEST has been suggested as a safer way to image glucose in vivo using exogenously given glucose before imaging^112^. Different viruses address the need for increased glucose differently, but in many cases, the result is an increase in the rate of glycolysis of infected cells^113^. Some notable viruses seen to increase glycolysis rates are SARS-CoV-2, MERS-CoV, DENV, HSV-1, and IAV^113^. Using glucoCEST could provide a non-invasive, non-radioactive method to monitor the inflammation mediated by these viruses.

CEST imaging of glutamate (Glu) is termed GluCEST and is sensitive to pH and concentration effects^111^. As Glu is one of the major neurotransmitters, levels of Glu in the brain can indicate a variety of pathologies. Glu is an important marker of neurotoxicity and damage for various pathologies and numerous viral conditions. Flaviviruses such as Japanese encephalitis virus (JEV) and West Nile virus (WNV), in particular, have been linked to changes in brain Glu concentrations^114^. Indications of increased Glu presence in brain regions that are not known to have naturally higher levels could be a sign of infection with a neurotropic virus. Decreased Glu levels can lead to what is known as glutamatergic dysfunction, which can cause cognitive deficits that include memory issues and attention deficits^115^. One case study of a SARS-CoV-2 patient experiencing cognitive dysfunction after the clearance of infection linked the virus to decreased glutamate in the prefrontal cortex^115^. Glu decrease has also been observed in HIV-related dementia^115^. In the case of SARS-CoV-2, the impacts of decreased Glu represent a putative causative factor involved in “brain fog” during and after infection and suggest possible treatments^115^.

## Discussion

### Possible future probes for viral imaging

There are many other possible viable MI probe targets for imaging viral infections that have yet to be tested. Ideally, targets would be unique to viruses rather than molecules or processes that are shared between the host and the pathogen. The best candidates for viral-specific imaging are enzymes or ligands that are required for viral-specific processes, such as viral proteases. Other options include probes activated by viral conformational changes during infection or those that target viral genome replication^16^. In short, examinations of the viral lifecycle can provide clues as to putative imaging targets. While constitutively expressed viral proteins are one option for imaging targets, transiently expressed proteins would also give insight into infection dynamics.

A big challenge in designing virus-specific probes is the reliance of viruses on host replication machinery. For example, certain viruses may encode their own polymerases for genome replication, while others will exploit those present in their host cells. Therefore, probes that work well for one virus will not necessarily be able to image a different virus specifically. Other viruses escape immune detection by coating themselves in host proteins, something that could also make their distinction from host cells by MI difficult. The viral mutation rate is also a factor to consider, with even probes targeting highly conserved proteins likely requiring updates to keep up with mutations. Often, new probes are suggested based on existing anti-viral therapies that have been seen to show low impact on host cells, indicating few off-target effects^16^. While many possible imaging options could be suggested, this section will discuss three possible novel probes targeting a different enzyme critical for the viral lifecycle and provide examples of how targeting would work in specific viruses.

### Imaging viral fusion

Viral fusion is critical in the lifecycle of enveloped viruses, allowing the viral membrane to merge with that of the host. Fusion peptides represent an attractive target for molecular imaging probes, as these peptides are highly specific to individual viruses or viral families. Prior to binding to entry receptors, the fusion proteins on viral membranes are in stable high-energy conformations. However, upon entry receptor binding, the fusion proteins undergo a conformational change, exposing a fusion peptide^116^. This peptide is usually hydrophobic and anchors the virus to the host for a short time. The fusion peptide conformation is not long-lived due to it being in an unstable energy state, eventually going into a post-fusion stable conformation where the viral and host membranes are merged^116^. Viral fusion proteins can be divided into three classes based on their structures, all of which have similar mechanisms, wherein binding to entry receptors or endosome acidification triggers a conformational change, exposing a fusion domain^117^. Several fusion inhibitors have been tested for different viruses, with these drugs being candidates for use as imaging probes.

In the case of SARS-CoV-2, its fusion peptide undergoes a conformational change upon attachment to host receptors^117^. This change results in the formation of a fusion core comprising two heptad repeats called HR1 and HR2. Critically, the binding of HR1 and HR2 can be prevented by peptide mimics of HR2^118^. These mimics have to bind HR1 similarly to the native subunit but result in a faulty fusion core. IAV has a similar fusion mechanism to SARS-CoV-2, with a subset of hemagglutinin called HA2 undergoing a pH-mediated conformational change^117^. Unlike SARS-CoV-2, mimics of the HA2 peptide do not result in defective fusion. Instead, IAV fusion inhibitors have been suggested to target conserved amino acids present in a hydrophobic binding pocket of HA2. These represent a viable anti-viral target due to their highly conserved nature amongst IAV subtypes^117^.

Fusion peptides represent an attractive possible target for molecule imaging probes. By adapting peptide mimics of HR2 to have a signaling moiety, it would be possible to image fusion of SARS-CoV-2. In the case of IAV, a probe would likely need to bind the conserved hydrophobic HA2 pocket to label the virus. The limitation with viruses such as IAV that enter via the endosomal route is the delivery of the anti-viral drug or MI probe into endosomes to interact with its target^117^. Solutions have been suggested for this issue, including the use of cell-penetrating peptides (CPPs) that show a high affinity for membrane interfaces. It is, therefore, possible to conjugate the probe to a CPP to aid in endosomal delivery^117,119^. The use of probes targeting fusion peptides for MI is further supported by the high specificity of these peptides to individual viruses or viral families. Due to the necessary conformational change to expose viral peptides, probes would only be able to bind during infection, providing a snapshot of viral infection dynamics.

Commenting on the chemistry required to manufacture these probes is beyond the scope of this paper. However, care must be taken not to alter the probe’s chemical characteristics due to the binding specificity. A more straightforward option would be using radio or fluorophore-labeled antibodies against the fusion peptides; however, due to the short length of the peptides, this has an increased risk of off-target effects. Fluorophores conjugated to the anti-fusion molecules are likely the best option, though due to the limitations of optical imaging, this would be mainly for preclinical use. The use of CEST is also promising as it may be able to detect exposure of the fusion peptide or binding of a mimic to it.

### Imaging viral helicases

Helicases are enzymes that are critical for nucleic acid replication^120^. In viruses, helicases have well-characterized roles in mRNA transcription, as well as the packing of viral genomes into newly made virions. Helicase inhibitors are a promising class of anti-viral drugs that have successfully reduced the replication of larger viruses. Adaptations of these drugs are therefore promising for directly labeling viruses for MI.

As helicases are shared across different kingdoms, they are by nature not necessarily virus-specific. This means that for specific probes to be designed, helicases that are common to humans and viruses should be avoided. Different viruses also use different replication strategies that determine which enzymes are used. Some viruses do not encode their own helicase enzymes but co-opt host helicases for replication. This is usually the case for smaller viruses that invade the host nucleus, as they have access to host enzymes and do not have the genomic space to encode their own. In contrast, larger viruses such as HSV and human papillomavirus encode almost all their required proteins^120,121^. While targeting viruses that use host helicases is highly unspecific, targeting viral helicases is a viable option for MI probes, as only infected cells will have this enzyme.

For HSV, two main helicases are involved in its replication, one of which complexes with other proteins to form the helicase-primase complex (HPI)^122^. This HPI is critical for the lifecycle of HSV and is conserved amongst the Herpesviridae viruses^122,123^. Several HPI inhibitors have shown promising results, reducing the growth of HSV without resulting in cytotoxicity for the host^121^. One thiazole-based inhibitor, amenamevir, has been seen to inhibit the replication of HSV and varicella zoster virus (VZV)^121,124,125^. While the mechanism by which amenamevir inhibits HPI of HSV and VZV is not well described, it has been approved in Japan for treating shingles and is well tolerated^125^.

Through modifications of amenamevir to include a signaling moiety, the drug can be adapted as an MI probe. The complex chemistry of attaching a signaling molecule without modifying its activity against HSV is left to the radiochemists. While the exact mechanism of action of amenamevir is hazy, it is known to directly inhibit the ATPase of the HPI, with mutants near the putative site of interaction with the virus conferring drug resistance. This suggests a binding action between the drug and the virus, making it suitable as an imaging candidate, though monitoring to ensure mutations have not altered binding would be required. The most likely signaling moiety for an amenamevir or other helicase inhibitor-based probes would be a fluorophore for use in optical imaging. Adding a standard organic fluorophore to the drug or using quantum dots could do this. QDs have been used to study other subcellular processes, including endocytosis of receptor-ligand complexes and transport of different cellular components within the cytoplasm^126^. Due to the resolution constraints of optical imaging, this probe may be best for use in preclinical research on animal models that have reduced tissue depth.

### CEST imaging of viral protease activity

A method of CEST imaging called catalyCEST can image enzyme catalytic activity^127^. CatalyCEST uses agents that contain amide bonds susceptible to cleavage by various proteases in the body. When this bond is cleaved, there is a chemical change in the aryl amide group to an aryl amine group that is not detectable with CEST. The activity of the protease can then be detected due to the loss of signal from the aryl amine group^127^. To improve image interpretation, these agents can include a second proton that can be exchanged and serve as a control. These different exchangeable protons can be distinguished based on their position on CEST Z-spectrums^127^. CatalyCEST agents have also been made that can detect enzyme-mediated covalent bond formation, sulfatase activity, and β-galactosidase activity^128–130^. A study using CEST to monitor the activity of caspase-3 noted that low concentrations of the enzyme could be detected and that the CEST agent showed specificity for caspase-3 over similar enzymes^131^.

CatalyCEST targeting the activity of proteases is of interest for the study of viral infection and pathogenesis. Proteases are characterized as targeting specific peptide bonds with defined targets^131^. Therefore, the specificity of proteases could be exploited in the design of catalyCEST agents, making the agents specific for proteases of interest. For example, Mpro is the main protease of SARS-CoV-2 and is highly specific for the virus, making it an ideal target for therapeutic interventions and an MI probe^132^. It is required to cleave the polyprotein produced during the replication of COVID-19 and is critical in the lifecycle of the virus. Furthermore, the structure and function of Mpro are conserved between coronaviruses, making it a target for the family of viruses. Mpro is a cysteine protease, a class successfully targeted by catalyCEST before^127^. Similar to the idea of the previously discussed SARS-CyCD probe, catalyCEST targeting the activity of Mpro could act as an activatable probe, allowing the localization of SARS-CoV-2 in the body.

The same principle could be applied to studying other viral families with similar enzymatic reactions. Another example is flaviviruses that have the highly conserved protease NS3^133^. Critically, it is thought that NS3 requires the presence of the membrane protein NS2B to be active, giving the enzyme increased specificity^133^. CatalyCEST agents that could be cleaved by the NS3-NS2B protease could act as imaging probes for many flavivirus family members.

## Conclusion

MI has historically been used more for cancer research than viruses; however, it has great potential for studying viral pathology. Several different MI technologies have been used with different types of probes to study viral pathology. These include direct monitoring of viruses and indirect probes that look at physiological changes induced by infection. Direct probes that can label viral proteins provide valuable information about the spread of the virus through the body and insights into the targeted tissues. Indirect probes, such as those that look at altered metabolism, indicate the immune response to infection. Monitoring the immune response informs not only on the host’s defense against the virus but also on possible virus-triggered immune pathology. Cancer probes can also be adapted to studying viruses. Many indirect probes used for the study of cancer are applicable to studying viral-induced pathology. This includes metabolic changes seen both in cancer and inflammation, as well as induction of cellular processes like apoptosis that are common to both pathologies. Other probes used in cancer, such as the HSV1 thymidine kinases, can be adapted from their original purpose and used for studying viruses. Other additional viral proteins could also be targets for future MI probes. The suggested direct probes target parts of the viral lifecycle that are key for infection and virus spread, thus giving valuable information about viral tissue tropism.

The potential of MI to be used in studying viral pathogenesis cannot be understated. The use of MI coupled with the probes discussed in this work stands to illuminate the pathogenesis of various viruses in vivo. The discussed probes can provide rapid, longitudinal, and non-invasive data pertaining to the spread of infection and the damage caused by it. The use of MI stands to benefit not only the study of known viruses but also of emerging pathogens. In the case of SARS-CoV-2, MI and the use of MI probes would have been able to provide data indicating that the pathology was not limited to the lungs faster than it was found through standard clinical investigation. With the strong likelihood of more novel pathogenic viruses emerging, all possible tools for evaluating viral pathogenesis must be used. This includes adapting existing probes and methodologies for the study of cancer to better prepare for new infectious disease threats.

While this work has discussed the extensive potential of using molecular imaging to study viruses, this is not to say that the prospect is without its challenges. Viral protein expression is often low level, meaning that there is a less abundant target for MI probes than in the case of imaging tumors. Furthermore, some viruses co-opt host processes and proteins for their lifecycle or to coat themselves and avoid the immune system, making the distinction between host and pathogen difficult. The high mutation rate in many viruses also poses a problem, as the targets of probes are likely to change over time. This can be mitigated through the selection of highly conserved targets, as well as genetic monitoring to inform any updates needed to the targeting of the probe. Imaging pathology induced by viruses also uses non-specific imaging, such as ^18^F-FDG, requiring more analysis and controls to distinguish viral induced from physiological signals. However, mitigation strategies are available for all these issues (discussed in the text) and should not prevent the use of molecular imaging in the study of viruses.

While the probes described here are largely for preclinical use, efforts should be undertaken to translate these imaging techniques into clinical practice. In particular, collaboration between clinicians and those designing probes would improve translatability and aid with intelligent probe design. Including virologists in this collaboration is paramount to adapting existing probes and designing novel ones to address viral threats and emerging pathogens. Using these probes in clinical settings could inform patient care, allowing for a minimally invasive method of assessing disease severity. Furthermore, treatment plans can be adjusted in response to the impact they have on the disease, with systemic monitoring of inflammation and metabolic changes giving insight into molecular-level changes not otherwise detectable.

## Data Availability

No datasets were generated or analyzed during the current study.
